# Role of the human supplementary eye field in the control of saccadic eye movements

**DOI:** 10.1016/j.neuropsychologia.2006.09.007

**Published:** 2007

**Authors:** Andrew Parton, Parashkev Nachev, Timothy L. Hodgson, Dominic Mort, David Thomas, Roger Ordidge, Paul S. Morgan, Stephen Jackson, Geraint Rees, Masud Husain

**Affiliations:** a Centre for Cognition & Neuroimaging, Brunel University, UK; b Institute of Cognitive Neuroscience, UCL, London, United Kingdom; c Division of Neuroscience and Mental Health, Imperial College, London, United Kingdom; d School of Psychology, University of Exeter, Exeter, United Kingdom; e Dept Medical Physics & Bioengineering, UCL, United Kingdom; f University of Nottingham, UK; g Institute of Neurology, UCL, Queen Square London, United Kingdom

**Keywords:** Supplementary motor area, Medial frontal cortex, Associative learning, Anti-saccades, Memory-guided saccades

## Abstract

The precise function of the supplementary eye field (SEF) is poorly understood. Although electrophysiological and functional imaging studies are important for demonstrating when SEF neurones are active, lesion studies are critical to establish the functions for which the SEF is essential. Here we report a series of investigations performed on an extremely rare individual with a highly focal lesion of the medial frontal cortex. High-resolution structural imaging demonstrated that his lesion was confined to the region of the left paracentral sulcus, the anatomical locus of the SEF. Behavioural testing revealed that the patient was significantly impaired when required to switch between anti- and pro-saccades, when there were *conflicting* rules governing stimulus–response mappings for saccades. Similarly, the results of an arbitrary stimulus–response associative learning task demonstrated that he was impaired when required to select the appropriate saccade from conflicting eye movement responses, but not for limb movements on an analogous manual task. When making memory-guided saccadic sequences, the patient demonstrated hypometria, like patients with Parkinson's disease, but had no significant difficulties in reproducing the order of saccades correctly on a task that emphasized accuracy with a wide temporal segregation between responses. These findings are consistent with the hypothesis that the SEF plays a key role in implementing control when there is conflict between several, ongoing competing saccadic responses, but not when eye movements need to be made accurately in sequence.

Animals that rely on visual information need to direct their eyes to behaviourally relevant aspects of a complex changing environment. This involves generating eye movements reflexively in reaction to sudden changes in the world (e.g. a saccade to an object suddenly appearing in the field of view) or using internally stored – endogenous – rules encoding arbitrary learned associations (e.g. obeying written instructions to look right). Both reflexive and endogenous saccades may need to be interrupted or modified on the basis of knowledge of the outcome of past performance, the aims of the organism and its current environmental context. Consequently, the generation of saccadic motor plans is an extremely complex behaviour linked to a wide variety of cortical and sub-cortical regions (Leigh & Zee, 2006).

In this study we focus on the role of the supplementary eye field (SEF), an area of the dorso-medial frontal cortex active during eye movements (Schlag & Schlag-Rey, 1987; Tehovnik, Somner, Chou, Slocum, & Schiller, 2000). Evidence from neural recording and stimulation studies in primates and functional imaging in humans indicate a potential role for the SEF in a wide range of saccadic tasks. These include learning arbitrary oculomotor stimulus–response rules (Chen & Wise, 1995a), reward or error monitoring (Amador, Schlag-Rey, & Schlag, 2000; Curtis, Cole, Rao, & D’Esposito, 2005; Stuphorn, Taylor, & Schall, 2000), encoding object-centred directions for saccades (Olson & Gettner, 1995), smooth pursuit (Heinen & Liu, 1997; Missal & Heinen, 2001, 2004; Tian & Lynch, 1995), self-paced eye movements (Petit et al., 1993), unpredictable sequential eye movements (Luna et al., 1998), antisaccades (Curtis & D’Esposito, 2003; Doricchi et al., 1997; Kimmig et al., 2001; O’Driscoll et al., 1995; Schlag-Rey, Amador, Sanchez, & Schlag, 1997) and the execution of memory-guided saccade sequences (Heide et al., 2001; Lu, Matsuzawa, & Hikosaka, 2002; Petit et al., 1993, 1996).

In general, these studies are suggestive of a role for the SEF in controlling ‘internally generated’ eye movements during the performance of complex learned behaviours (Guitton, Buchtel, & Douglas, 1985; Leigh & Zee, 2006), but it remains unclear which specific aspects of oculomotor behaviour critically involve the SEF. A few studies have attempted to determine the function of the SEF by examining failures in saccadic performance of patients with lesions subsuming this area (Braun, Weber, Mergner, & Schulte-Monting, 1992; Gaymard, Pierrot-Deseilligny, & Rivaud, 1990; Gaymard, Rivaud, & Pierrot-Deseilligny, 1993; Heide, Kurzidim, & Kompf, 1996). Unfortunately, patients in these studies had large lesions involving other cortical areas, so any reported deficits cannot unequivocally be associated with the SEF.

In the current study, we report data from an extremely rare patient with a highly focal lesion of the left SEF across a range of saccadic tasks, which allows a unique opportunity to specifically identify tasks in which the SEF plays a critical role. Previously, in a brief communication, we showed that this patient had great difficulty changing from an initial saccade plan to an alternative one using a novel change-of-plan paradigm. We also found that he experienced difficulty when he was required to reverse a rule regarding the direction of saccade cued by a stimulus (Husain, Parton, Hodgson, Mort, & Rees, 2003). These effects were bilateral, consistent with the known bilateral representation of saccade directions in each SEF (Tehovnik et al., 2000). Subsequently, functional imaging of healthy individuals performing a change-of-plan saccadic task demonstrated enhanced SEF activity when subjects were successful at changing their saccadic plans (Nachev, Rees, Parton, Kennard, & Husain, 2005). On the basis of these data we proposed that a major role of the SEF lies in implementing control over conflicting internally generated saccadic plans (Husain et al., 2003; Nachev et al., 2005).

One attractive aspect of this hypothesis is that it may help to provide a unifying account for many of the disparate findings regarding SEF function. The aim of the current study was to further characterize the role of the SEF in oculomotor control, particularly with respect to its proposed function in resolving competition between conflicting saccadic plans. In order to do this we first present new high-resolution structural imaging that shows the precise extent of the lesion in axial and sagittal planes. These images demonstrate that the lesion is limited to the SEF and, crucially, does not encroach on the anterior cingulate cortex, a nearby brain region that has been proposed to play a role in conflict *detection* (Braver, Barch, Gray, Molfese, & Snyder, 2001).

In our first set of behavioural experiments we examined the patient's performance when required to saccade either directly towards or away from a target that suddenly appeared in his visual field, respectively, termed pro- and anti-saccades (Munoz & Everling, 2004). The anti-saccade task necessitates the resolution of conflicting reflexive and rule-based saccadic plans and electrophysiological studies in monkeys have implicated the SEF in their production (Amador et al., 2000; Amador, Schlag-Rey, & Schlag, 2004; Schlag-Rey et al., 1997). However, the exact nature of the SEF's role remains unclear as the production of an anti-saccade involves two major components: suppression of a reflexive pro-saccade towards the target *and* the application of a learned arbitrary rule to generate a saccade in the opposite direction (Munoz & Everling, 2004; Olk, Chang, Kingstone, & Ro, 2005). Furthermore, the physiological evidence is based on a paradigm in which pro- and anti-saccades were intermingled in ‘mixed blocks’ so neural activity might also reflect systems used to switch behavioural rules on a trial-by-trial basis. In our experiments, we sought to examine whether damage to the SEF lead to a difficulty suppressing pro-saccades, executing an arbitrary behavioural rule or switching between rules, i.e. when it was necessary to resolve competition between two conflicting rules for saccades.

In our second set of experiments we examined the proposed role of the SEF in learning arbitrary oculomotor stimulus–response mappings by trial and error (Chen & Wise, 1995a, 1995b, 1996, 1997). Specifically, we examined whether any impairment was attributable to a difficulty suppressing previously learned stimulus–response associations, or selecting from *competing* alternative potential responses. Furthermore, because our previous imaging data (Husain et al., 2003) led us to conclude that JR's lesion involved the SEF but not the hand area of the supplementary motor area (SMA), we also examined learning of stimulus–response associations for eye versus hand movements. If the deficit is, as we expect, a result of problems selecting or reinforcing an appropriate *motor* response we predicted that it would be specific to the saccadic system following a lesion restricted to the SEF. In contrast a more general difficulty learning the meaning of a stimulus would affect performance in both eye and hand tasks.

In our final set of experiments we examined JR's performance when making either single or sequences of eye movements to remembered locations. Previous studies have implicated the supplementary motor areas generally in the ordering of motor sequences and the SEF, specifically, in ordering series of saccades (Isoda & Tanji, 2002, 2003; Mushiake, Inase, & Tanji, 1990; Tanji & Shima, 1996). Furthermore, two reports of Gaymard et al. (1990, 1993) have reported that lesions incorporating specifically the *left* SMA lead to difficulty in ordering memory-guided saccades. In theory, such a deficit might arise from a difficulty resolving conflict between alternative responses segregated over time. However, this ordering deficit might not be attributable to damage to the SEF itself but might instead result from damage to neighbouring regions also known to play a role in processing sequential motor plans, e.g. the pre-supplementary motor areas (pre-SMA) (Clower & Alexander, 1998; Nakamura, Sakai, & Hikosaka, 1998). So it is important to explore the effects of focal lesion of the SEF on memory-guided saccadic sequences. In addition, we wanted to compare performance on memory-guided sequences with *single* memory-guided saccades. If both types of eye movement were impaired in the same way, it would suggest that the SEF may not have a special role in sequencing eye movements but instead may play a role in generating the appropriate response to a stimulus that is no longer visible, following an intervening delay.

## 1 General methods

### 1.1 Patient details and lesion localization

Patient JR is a right-handed man who was 55 years old when he suffered an extremely small left medial frontal venous stroke. On clinical examination, 8 months after the stroke and at the time these experiments were conducted, there were no longer any abnormal physical signs. There was no evidence of aphasia, apraxia or visuospatial deficit. Forward digit span was 7, and on the Mini-Mental State Examination (Folstein, Folstein, & McHugh, 1975), he scored 30/30. On neuropsychological tests considered to be sensitive to frontal executive control he performed within normal limits, e.g. he was between the 25th and 50th percentile on perseverative errors using the Wisconsin Card Sorting Test (Nelson, 1976), in the 19th percentile on the Stroop test (Stuss, Bisschop, Alexander, Levine, & Katz, 2001) and between the 25th and 50th percentile in Trail Making (Arbuthnott & Frank, 2000).

**Fig. 1:**
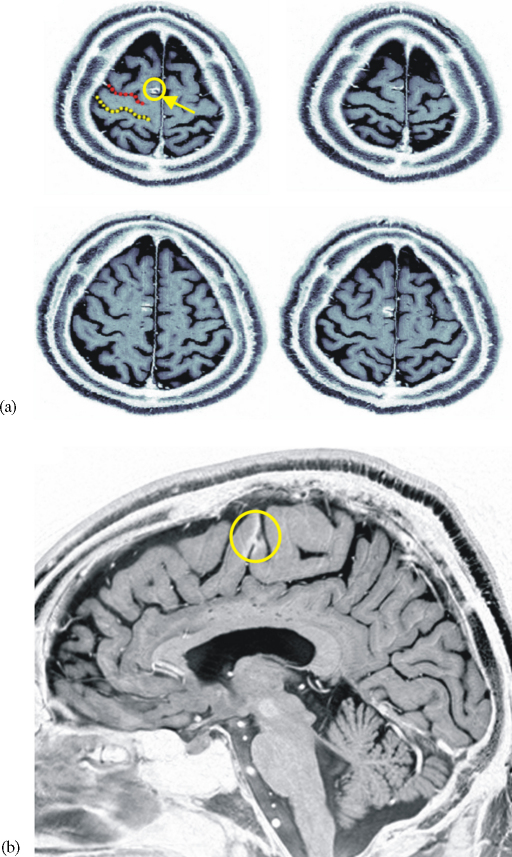
High-resolution structural MRI images presented with the grey scale inverted showing four axial slices (a) and a saggital slice (b) acquired on a 4.7 and 3 T scanner, respectively. The extent of venous infarction can be seen by the small area of signal change in (i) the left hemisphere on the axial slices and (ii) the dorso-medial area on the saggital image. On the axial slices, the precentral sulcus (red dots) and central sulcus (yellow dots) are marked on one slice, together with the paracentral sulcus (green arrow)—the anatomical landmark of the human SEF.

To clearly demonstrate the anatomical locus and extent of JR's lesion we acquired two new sets of high-resolution structural MRI scans (Fig. 1a and b) showing the position of the lesion on both axial and sagittal planes, which considerably improve on the in-plane resolution of the previously published structural scan (Husain et al., 2003). The new axial structural images were acquired using 2D Fast Spin Echo (FSE) imaging performed on a 4.7 T whole body scanner (De Vita et al., 2003), and the sagittal image was acquired on a 3 T scanner. The advantage of using high field systems (i.e. greater than the standard clinical field strength of 1.5 T) is the improvement in image signal-to-noise, which can be used to increase spatial resolution while still maintaining good image quality. For the 4.7 T scan, the spatial resolution of the images was 0.47 mm × 0.47mm (in plane) × 2 mm (slice thickness), obtained using a 512 (read) × 776 (phase encode; 8 echoes per shot; oversampled ×2) acquisition matrix. A nominal echo time of 66 ms was chosen to achieve T_2_-weighting; repetition time was 3.5 s, sufficient to obtain 17 slices (scan time was 5 min 40 s). For the 3 T scan, a T_2_ weighted Turbo Spin Echo (TSE) sequence was used (TE = 80 ms, TR = 3000 ms, echo train length (ETL) = 15, 4 averages, acquisition matrix 400 × 400, pixel size 0.58 mm × 0.58 mm). Twenty-four sagittal slices were acquired with slice thickness of 1.5 mm with 10% slice gap, in two interleaved packages (acquisition time was 10 min).

The new scans clearly demonstrate that JR's lesion is restricted to the area of the left paracentral sulcus (Fig. 1a and b), the location that is considered to be the anatomical landmark of the human SEF (Grosbras, Lobel, Van de Moortele, LeBihan, & Berthoz, 1999). Importantly, the lesion does not extend into the cingulate cortex, a region that has been implicated in conflict detection. In our previous brief communication, we reported functional imaging localizer data showing that activation of the SEF in the undamaged right hemisphere occupied the region immediately opposite the lesion. In addition, activity during a go/no-go finger movement protocol indicated that the hand representation in the left SMA was intact (Husain et al., 2003). Taken together with the new structural imaging presented here these data demonstrate that Patient JR has a highly localized lesion of the left SEF.

### 1.2 Apparatus and stimuli

In all experiments, unless noted otherwise, eye movements were recorded using a video-based pupil tracker with a temporal resolution of 4 ms and spatial accuracy of <0.5° (EyeLink; Sensorimotoric systems GMbH, Berlin, Germany). The stimuli were presented on a uniform grey background on a 21-in. CRT monitor (Iiyama, Japan).

## 2 Experiment 1: Pro- and anti- saccades

The first experiment was a detailed examination of JR's performance across a series of pro- and anti-saccade tasks. Specifically we examined the effects on three components of the anti-saccade task: (i) suppression of an automatically generated reflexive saccade, (ii) execution of a saccade in the opposite direction and (iii) the ability to flexibly switch between conflicting rules that link the specific required saccadic behaviour to the stimulus.

First, we investigated whether JR had a problem suppressing the impulse to make a reflexive saccade by examining the effect of introducing a gap between the disappearance of the fixation cross and the onset of the saccadic cue. In normal participants, when ‘gap’ conditions are compared to those without a gap (between fixation offset and cue onset) there is an increased tendency to reflexivity, which is apparent in decreased pro-saccade reaction times and increased anti-saccade direction errors (Munoz & Everling, 2004). If JR has particular difficulty cancelling a reflexive (pro-saccadic) impulse we might expect that the introduction of a gap would produce a disproportionately increased number of anti-saccade direction errors.

Second, we assessed whether JR has a basic problem implementing a behavioural rule to execute an anti-saccade (i.e. suppress a reflexive glance and implement an internally generated saccades), or whether he encounters problems swapping between potentially conflicting behavioural rules governing the stimulus location and response direction. To isolate the latter effect, we examined his performance in mixed blocks of pro- and anti-saccades (where he has to swap between behavioural rules that govern the correct response to a stimulus onset) and separate (pure) blocks of each task which remove the need constantly to change stimulus-behaviour mappings (Hallett & Adams, 1980; Hodgson, Golding, Molyva, Rosenthal, & Kennard, 2004). JR should have exhibited a deficit in the mixed task compared to separate (pure) task blocks if the SEF plays a critical role in swapping between conflicting behavioural rules.

### 2.1 Methods

Each participant performed six randomly ordered experimental blocks comprising one of three possible task combinations: (i) pro-saccades only, (ii) anti-saccades only and (iii) evenly mixed pro- and anti-saccades (presented in a random order). Participants were informed before each block whether they would be making pro-saccades, anti-saccades or a mixture of tasks. On an individual trial the instruction to make a pro- or anti-saccade was signalled by the fixation cross colour (either black or white) with the relationship between colour and response counterbalanced across participants. There were six controls participants with a mean age 57.8 years (S.D. 3), who each completed 40–80 trials in every single task condition and 80–160 trials for all mixed task conditions. The controls for this, and all subsequent, experiments were selected from two lists of volunteers previously compiled by the Institute of Cognitive Neuroscience (UCL) and the Division of Neuroscience and Mental Health (Imperial College) from respondents to advertisements in adult education centres, Charing Cross hospital and the press. They all had some previous experience of participating in behavioural experiments with eye tracking but were naïve to the current hypothesis. None had any history of neurological conditions. JR performed 200–400 trials in the each of the single task conditions and 600 trials in both the mixed conditions.^1^ Trials from all conditions were performed across three experimental sessions (occurring between 2 and 3 years after his stroke).

**Fig. 2:**
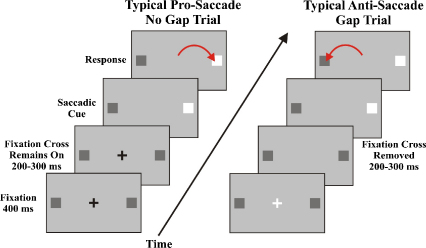
An illustration of the time course of typical pro- and anti- saccade trials. Subpart (a) shows a pro-saccade without a gap and (b) depicts an anti-saccade with a gap between the disappearance of the fixation cross and the eye movement cue. Participants fixated a central cross for 500 ms with its colour signalling either a pro- or anti-saccade trial. This was followed by a 200–300 ms delay period with (no gap condition) or without (gap condition) the fixation cross present. A saccade was signalled by a change in colour (grey to white) of either the left or right target square and participants were instructed to respond as rapidly as possible.

Fig. 2 illustrates the basic timeline for a typical pro-saccade trial without a gap (Fig. 2a) and an anti-saccade trial with a gap (Fig. 2b). Trials started with the presentation of a central fixation cross (1.83° long × 0.52° wide), flanked by two peripheral dark grey squares (1.31° across) to the left and right of centre (eccentricity 9.25°). When the participant had continuously fixated the central location for 400 ms the fixation cross was either removed (gap) condition or remained unchanged (no-gap). After a further 200–300 ms (determined randomly) a saccade was cued by one of the peripheral squares changing in colour to white (and the removal of the fixation cross in the no-gap condition). In pro-saccade conditions participants were instructed to look as quickly as possible at the white square and in anti-saccade conditions they were told to look at the opposite (unaltered) box.

### 2.2 Results and discussion

**Fig. 3:**
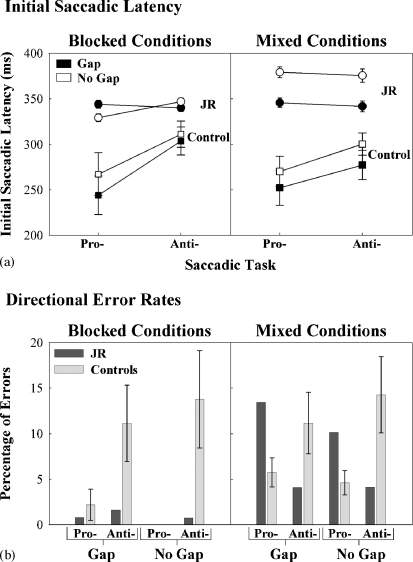
The figure shows the reaction time (a) and direction error (b) data for pro- and anti-saccade tasks. In (a) the primary saccadic latency of both JR (circles) and control participants (squares) is plotted for single (left graph) and mixed (right graph) task conditions. The fixation condition is indicated by filled (gap) and unfilled (no gap) shapes. In (b) the error direction rates for single (left) and mixed (right) task conditions are shown on two histograms. In each histogram the data are subdivided by fixation condition (gap versus no gap), task type (pro- or anti-saccade) and subject (JR or controls). All error bars indicate the S.E.M.

Fig. 3 depicts, for JR and the control participants, the mean latency of their primary saccades in correct trials (Fig. 3a) and the percentage of direction errors (Fig. 3b). Preliminary analysis revealed that neither control participants nor JR demonstrated lateralized differences in performance for any of the tested conditions (*p* > 0.1) and so this factor was excluded from all further analysis. To confirm the validity of the experimental manipulations data from the controls for both measures was initially analyzed using ANOVAs with the task type (pro-saccade versus anti-saccade), fixation condition (gap versus no-gap) and block (mixed versus single) as factors. For latency, there were main effects of task type (*F*_1,6_ = 11.33, *p* < 0.05) and fixation condition (*F*_1,6_ = 7.27, *p* < 0.05) but no effect of block nor any interactions (*p* > 0.1). The control participants made significantly more anti-saccade direction errors than for pro-saccades (*F*_1,6_ = 7.38, *p* < 0.05) but showed no other significant differences in their error rates. Taken together these results confirmed that normal participants showed a cost for anti-saccades in comparison to pro-saccades and for no-gap against gap conditions (Fischer & Weber, 1992; Machado & Rafal, 1992; Munoz & Everling, 2004).

A comparison of JR's reaction times in the pro- and anti-saccade tasks with those of the control participants indicated that he was significantly slower at both tasks (Fig. 3a). The increased latency was greater for the pro-saccade task, which was on average 91 ms (S.D. 20) larger than for controls, and this was significant across all four conditions (single gap: *t*(6) = 4.72, *p* < 0.005, single no-gap: *t*(6) = 2.79, *p* < 0.05, mixed gap: *t*(6) = 4.83, *p* < 0.005, mixed no-gap: *t*(6) = 6.59, *p* < 0.001). For anti-saccade conditions JR's saccadic latency was on average 53 ms (S.D. 20) larger than for controls, which was significantly different from chance in three task conditions (single gap: *t*(6) = 2.5, *p* < 0.05, mixed gap: *t*(6) = 4.02, *p* < 0.01, mixed no-gap: *t*(6) = 6.07, *p* < 0.001) and showed a strong trend towards significance in the fourth (single no-gap: *t*(6) = 2.37, *p* < 0.06). Most importantly, for JR there was no significant difference in latency between pro- and anti-saccades. The mean difference in his reaction times between the two tasks collapsed across all four experimental viewing conditions (single gap, single no gap, mixed gap and mixed no gap) was only 1.5 ms (S.D. 6.9 ms).

Any interpretation of JR's increased reaction times needs to consider also his directional error rates (Fig. 3b). These suggested that the increased latencies were either a consequence of, or resulted in, a speed-accuracy trade-off (i.e. longer latencies are associated with fewer errors). Crucially, in the single task conditions (i.e. pure blocks of either pro-saccades or pure blocks of anti-saccades) JR made virtually no errors in either pro- or anti-saccade conditions. In fact, on the anti-saccade task, he was actually *better* than controls, making far fewer errors (significant in the no-gap condition (*t*(6) = 2.44, *p* < 0.05) and showing a trend towards significance in the gap condition (*t*(6) = 2.27, *p* < 0.1). Similarly, in blocks where the two tasks were mixed he showed a trend towards making fewer errors than controls in the anti-saccade task (mixed gap: *t*(6) = 2.1, *p* < 0.1, mixed no-gap: *t*(6) = 2.42, *p* < 0.1). The relatively low error rates in anti-saccade tasks and the absence of any effect of a gap between fixation onset and target offset indicated that JR could impose saccadic control to perform the two basic components of this task: cancelling a reflexive saccade and implementing an arbitrary rule to determine saccadic behaviour.

The most important findings from these experiments comes from the examination of the error rates on *mixed* blocks of trials where participants had to switch between pro- and anti-saccades (Fig. 3b). Under these conditions, JR again performed well compared to healthy control individuals when cued to make anti-saccades (mixed gap: *t*(6) = 2.1, *p* < 0.1, mixed no-gap: *t*(6) = 2.42, *p* < 0.1). However, he was significantly *worse* than controls when required to make pro-saccades (mixed gap: *t*(6) = 4.82, *p* < 0.01, mixed no-gap: *t*(6) = 4.88, *p* < 0.01). Thus, when the cue signalled a pro-saccade, he was more likely than normal participants to make an anti-saccade. Note that this occurred despite his having significantly longer latencies for pro-saccades than controls (Fig. 3a), and demonstrating virtually no difference between his pro- and anti-saccades latencies (Fig. 3a). So a speed-accuracy trade-off cannot easily account for this specific deficit on pro-saccades in the *mixed* block condition.

A more detailed examination of the error rates revealed that significantly more of JR's pro-saccade direction errors (81%) occurred following an anti-saccade trial (i.e. after a rule switch). Furthermore, he was four times more likely to make an error when switching from an anti-saccade to a pro-saccade than when switching the other way around. The increased error rate when he was required to switch between anti- and pro-saccades provides further evidence for the potential importance of the SEF for resolving conflict between competing alternative responses (Husain et al., 2003). The data do not suggest that JR has a specific deficit in executing anti-saccades. Although he was slightly slower than controls, he was consistently *more* accurate. Therefore, these findings indicate that the left SEF is not essential to implement arbitrary behavioural rules to generate saccades; nor is it necessary to cancel reflexive saccades. By contrast, it does appear to play a role when required to switch between conflicting stimulus–response rules for saccades.

## 3 Experiment 2: Learning stimulus–response associations for eye and hand

An important aspect of behaviour is the ability to acquire new stimulus–response mappings by identifying, remembering and selecting the appropriate response to make in a particular environmental context. Experimentally, the learning of stimulus–response associations can be examined by asking individuals to establish, by trial and error, the appropriate response for a given stimulus from a range of alternatives; see, for example, Chen and Wise (1995a, 1995b, 1996, 1997) for studies on the macaque SEF neurones during learning of arbitrary stimulus–response associations. These paradigms involve the selection of an action amongst competing alternatives and the reinforcement of the stimulus–response linkage via positive feedback (or its inhibition following negative feedback). In principle, errors might occur in such a paradigm from a general failure to encode the meaning of stimuli. An alternative mechanism would be difficulty in resolving competition between possible conflicting responses when selecting a motor plan. One way to distinguish between these alternatives is to examine performance using different effector systems. A general difficulty in learning rules might be expected to be apparent regardless of the effector system – eye or hand – whereas a problem in response selection might be limited to a specific effector. In the current study, therefore, we contrasted performance for JR and age matched controls in closely matched associative learning tasks in which a response was made either manually or with the eyes.

### 3.1 Methods

**Fig. 4:**
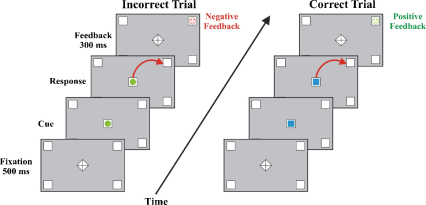
A schematic representation of typical trials in the saccadic stimulus–response association task. A fixation cross is followed by the appearance of colour/shape cue and participants respond by moving there eyes to one of the four place markers. They then receive positive or negative feedback to indicate whether that is the location associated with the cue, correct response feedback is illustrated on the right and incorrect on the left.

In the oculomotor version of the task, participants had to learn, by trial and error, the correct saccades to make in response to each one of four visual stimuli presented centrally on a Sony 15.1” TFT screen. The display was replaced by a central diamond outline (2.5°) with a black fixation cross within it, and four peripheral square boxes (2.5°) in each of the four corners of the screen (eccentricity 13°). These four peripheral boxes were the potential locations to which the subjects could saccade (Fig. 4). After 50 ms, the central diamond was replaced by a randomly selected instructional stimulus—an arbitrary coloured shape that was uniquely defined by both colour and form. The stimulus was selected randomly from a set consisting of a blue square, a green circle, a red cross or a yellow triangle, and the same stimulus set was used across all experimental blocks.

Participants were required to saccade to the peripheral box, which they believed to be associated with the current instructional stimuli. They received immediate feedback to indicate if their choice was correct or incorrect in the form of a happy/sad face icon (denoting, respectively, correct/incorrect responses) displayed within the selected box. After 400 ms, all of the elements on the screen were erased. Of course, at the beginning of each experimental block, subjects would have to guess which saccade might be appropriate for a particular coloured shape. However, using the feedback given, they could through trial and error, establish the correct stimulus–response mappings for each of the central cues. Each experiment block terminated when the participant successfully reached a criterion of correctly performing 11 trials consecutively. The subject was informed a new block would begin, with the computer randomly re-assigning each of the central coloured shapes to be associated with saccades to a particular peripheral box, i.e. generating a new set of S-R mappings.

In the manual version of the task, participants were required to make pointing movements using the right hand rather than saccades. This paradigm followed a procedure closely matched to the saccadic task with the following exceptions: there was only a small separation (3°) between the peripheral boxes and the instructional stimulus so that subjects did not make saccades to the locations they reached to (If they had made eye movements as well as hand movements, this would not have been a pure test of S-R rule learning for manual movements). Participants made their response by reaching to press the appropriate box on a touch sensitive screen. They rested their hand on the desk on which the touchscreen stood between responses. Stimuli were displayed on an NEC MultiSync LCD2010X 21” TFT monitor with a capacitive touch screen sensor (Mass Multimedia, Inc., Colorado Springs, USA). All participants performed one practice block in each modality before beginning. The results for each of the four controls (mean age 58 years; S.D. 5) reflect mean performance across six to eight blocks for each response modality performed in one experimental session. Patient JR completed 12 blocks in each modality (with six blocks of each response type in two experimental sessions).

### 3.2 Results and discussion

**Fig. 5:**
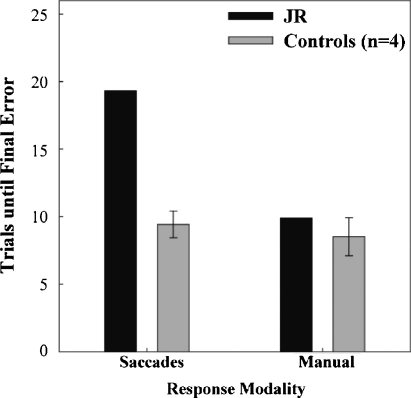
The number of trials that each participant required to achieve criterion (11 consecutive error-free trials) on both stimulus–response associative learning tasks, with error bars representing the S.E.M.

Fig. 5 depicts the number of trials participants took to reach stable error-free performance (criterion = 11 consecutive correct trials) in both the eye movement and manual tasks. On the oculomotor task, JR was severely impaired, requiring significantly more trials to learn the stimulus–response association than the control participants (19 trials versus 9 trials), with the difference in performance between controls and JR being >5S.D.s, where the 99% probability level is ∼2.6S.D.s (two-tailed).

By contrast, JR's performance on the manual task was almost indistinguishable from that of the control participants (i.e. <1S.D. from the mean). Thus, while control participants demonstrated almost identical performance in both response modalities (saccadic and manual), JR was significantly worse for the eye movement task than the manual task (Wilcoxin's Signed Ranks *T* = 45, *p* = 0.004). The results cannot be explained by an increased error rate resulting from a lateralized response bias: JR showed little differences in left versus right errors in either saccadic (46% left) or manual tasks (43% left).

One possible explanation for JR's difficulties in the oculomotor task is that they reflect an intrusion of the association mapping learned in the previous block of trials. However, a careful analysis of the data revealed no evidence to support the contention that his responses reflected perseverance from the mapping learned in the previous block. The percentage of his erroneous responses (28%) made to targets corresponding to the previous mapping was almost exactly the same as that for control participants (mean 27%; S.D. 8%). Similarly, an analysis of the first response that the participants made in each block suggested that JR was no more likely to respond on the basis of the previous mapping than controls (16% versus 22% (18% S.D.)).

In summary, the results of this experiment showed that JR has difficulty on the oculomotor task, but he was normal in a closely matched manual task indicating that he does not have a generalized difficulty in learning stimulus–response associations. Rather, the data are consistent with a difficulty selecting a weakly reinforced rule (i.e. one which has received correct feedback but is not yet well established) in the face of conflicting alternative responses, but only for eye movement responses (Husain et al., 2003).

## 4 Experiment 3: Memory-guided saccades

In our final experiment we examined JR's behaviour in a task that required the generation of *single* memory-guided saccades or memory-guided *sequences*. In the latter situation the participant was required to store and recover several locations, or movement plans, in the correct order. Previous studies of individuals with lesions subsuming the SEF have reported order errors in a memory-guided saccade sequences for patients specifically with *left* SEF damage (Gaymard et al., 1990, 1993; Lu et al., 2002). In theory, this might also reflect a difficulty in selecting between competing eye movement plans *over time*. However, these patients all had lesions that extended into other adjacent motor areas, which are also considered to be important in motoric sequencing (i.e. the pre-SMA, see Clower & Alexander, 1998; Nakamura et al., 1998). Therefore, we wished to examine whether focal damage to the left SEF leads to errors reproducing the correct order of memory-guided saccade sequences.

### 4.1 Methods

**Fig. 6:**
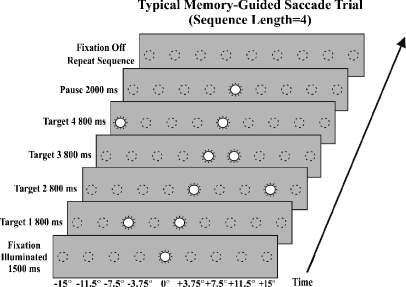
A schematic representation of typical trials in the memory-guided saccades task**.** Participants maintained fixation on a central LED whilst a sequence of successive lights were selected at randoml from either of fixation and illuminated. Two seconds after the final light in the sequence the fixation light was removed, which cued participants to execute eye movements to each sequence location (in the correct order). The figure illustrates a typical right sided sequence. Illuminated LEDS are indicated by filled white circles with lines radiating from them and the position of unlit LEDs is indicated by broken circles.

Memory-guided sequences were tested using a protocol illustrated in Fig. 6 and described previously (Hodgson, Dittrich, Henderson, & Kennard, 1999). Participants fixated a central illuminated LED at the beginning of each trial. After 1500 ms, four LEDs were selected at random from eight potential locations (3.75°, 7.5°, 11.25° and 15° either side of the fixation light) and illuminated in succession for 800 ms. Participants had to maintain central fixation throughout the sequence and for a further 2 s. After this the central LED was extinguished, and participants were required in the dark to reproduce the sequence by making saccades to the memorized locations in the correct order. Participants were asked to match the timing intervals between the viewed stimuli so that they maintained a brief fixation at each stimulus location. There were 25 sequences in each experimental condition and all participants also completed 10 practice trials.

Additionally, participants performed a control task making sequences of saccades to targets that remained illuminated. Eye movements were recorded using an infrared limbus tracker with a temporal resolution of 2 ms and spatial accuracy of <0.5° (Skalar IRIS). There were 12 controls (mean age 68 years; S.D. 3). Finally, we also examined performance of memory-guided saccades to single targets. This task acted as a second control and extremely important baseline task. If performance on the sequence task could be predicted entirely by performance on the single memory-guided saccade paradigm, it would suggest that the SEF does not play a specific role in the generation of memory-guided saccadic sequences.

### 4.2 Results and discussion

**Fig. 7:**
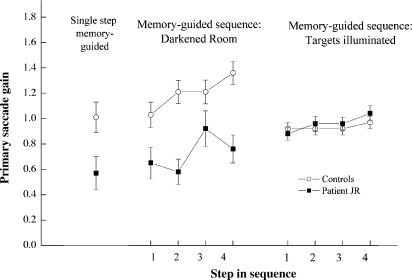
Performance of memory-guided saccades. The graphs depict the primary gain for saccades to a single remembered location (left), and for each step in a four target sequence when the targets were removed (centre) or left illuminated (right). Error bars indicate the S.E.M. JR's saccades (black squares) are clearly hypometric in comparison to the control participants (white circles) when location markers are not present for one, or more, remembered locations.

In contrast to previous studies of patients with – less focal – lesions involving the left SEF (Gaymard et al., 1990, 1993), JR did *not* make significantly greater errors on four-step memory-guided sequences in the dark (21% sequence recall errors cf. 39% (±5%) for controls). Thus, he did not demonstrate any impairment in generating the correct *order* of saccade sequences. The only deficit demonstrated by him for memory-guided saccades was a marked hypometria in his primary saccade gain (see Fig. 7), which was evident for *both* single and sequences of memory-guided saccades.

On single memory-guided saccades to remembered targets, primary saccadic amplitudes were also significantly reduced (gain = 0.44 left versus 0.70 right) compared with controls (means = 1.03 versus 1.00 with corresponding lower 95% limits being 0.79 and 0.83, respectively). Final eye position demonstrated an undershoot, again more marked to the left than the right (gain = 0.53 left versus 0.83 right, *t* = 2.33 *p* < 0.05) compared with controls (means = 1.38 versus 1.34; lower 95% limits: 1.08 and 1.15, respectively).

For saccadic sequences, primary saccade amplitudes were also significantly reduced (mean primary gain left = 0.66 versus 0.76 right) compared with controls (means = 1.06 and 1.04; lower limit of 95% confidence intervals = 0.82 and 0.87, respectively). However, final eye position on this task, in which controls usually overshoot the target location, was crucially within normal limits, although leftward amplitudes were still significantly smaller than those to the right (means = 0.95 and 1.25 for left and right saccades, *t* = 2.34 *p* < 0.05; corresponding 95% confidence intervals = 1.07–1.70 and 1.15–1.52). Importantly, JR's primary saccade hypometria was not observed in the control condition where he was asked to make saccadic sequences to targets that remained continually illuminated (Fig. 7).

In summary, JR demonstrated no difficulty in correctly ordering responses for memory-guided sequences, which indicates that the left SEF is not required to resolve any potential conflict between eye movement plans when they are temporally segregated as on this task. The only deficit JR exhibited was on memory-guided sequences and single memory-guided saccade tasks where his saccades undershot target locations.

## 5 General discussion

The findings reported here describe the results of imaging and behavioural investigations conducted on JR, an extremely rare individual who has a highly selective lesion of the medial frontal cortex. The new high-resolution structural MR imaging studies performed at 4.7 and 3 T demonstrate that JR's lesion is highly focal (Fig. 1), located at the left paracentral sulcus, the known anatomical landmark of the SEF (Grosbras et al., 1999). The lesion does not extend more ventrally into the cingulate cortex, but rather is confined to the medial superior frontal gyrus. Taken together with previous functional imaging localizer studies for eye and hand movements (Husain et al., 2003), these new structural imaging results provide strong evidence that his lesion selectively involves the SEF.

The new behavioural results presented here demonstrate that a selective lesion of the left SEF leads to difficulties in switching from anti- to pro-saccades (Fig. 3), i.e. when there are *conflicting* rules to select from regarding stimulus–response mappings for saccades. Similarly, the results of the arbitrary stimulus–response learning task (Fig. 4) revealed that JR took longer to reach criterion than healthy controls when required to select the appropriate saccade from conflicting possible alternative saccadic responses (Fig. 5). This did not occur when he was required to select the appropriate limb movement from competing alternatives in analogous manual task (Fig. 5), so he does not appear to have a general problem in understanding the task, encoding the stimuli or simply learning. These findings would be consistent with the hypothesis that the SEF plays a key role in resolving conflict between competing saccadic responses (Husain et al., 2003). Such a proposal also accords well with the results of a recent electrophysiological study in macaques which reports increase SEF activity in situations of saccadic response conflict (Nakamura, Roesch, & Olson, 2005).^2^

Previous studies have suggested that the left SEF, in particular, may have a key role in encoding the correct order of saccades when making eye movements to remembered positions (Gaymard et al., 1990, 1993). Our study did not find such a deficit, but instead observed that both memory-guided sequences and single saccades demonstrated hypometria when made in the dark, but not when all target lights were kept illuminated (Fig. 7). The hypometria is discussed further below, but the lack of a deficit in ordering saccadic sequences suggests that the left SEF is not required to resolve potential conflict between eye movement plans when they are temporally segregated and have to be made *in sequence*. Rather, the critical role of the SEF may be best demonstrated when ongoing saccadic plans suddenly have to be altered (Husain et al., 2003) or the association between a stimulus and the saccadic response has to be changed (as when switching from anti- to pro-saccades) or when selecting the appropriate saccade from a set of competing possibilities when the stimulus–response associations are weakly reinforced (as in the arbitrary stimulus-learning task).

### 5.1 Anti-saccades and the control of internally-generated saccades

Previous electrophysiological studies in monkeys have implicated the SEF in the control of anti-saccades (Amador et al., 2000, 2004; Schlag-Rey et al., 1997). However, the precise contribution of the SEF to such control has not been resolved. The production of an anti-saccade involves both the suppression of a reflexive pro-saccade towards the target *and* the application of a learned arbitrary rule to generate a saccade in the opposite direction (Munoz & Everling, 2004; Olk et al., 2005). The paradigm used in macaques to study SEF involvement in anti-saccades intermingled pro- and anti-saccades in ‘mixed blocks’, so neural activity might potentially reflect systems used to switch behavioural rules on a trial-by-trial basis.

In the experiments reported here, when a behavioural rule was clearly established and did not alter (as in blocks of pure anti-saccades), JR performed extremely well. In fact, he made significantly fewer errors than healthy controls (Fig. 3). Thus, seemingly paradoxically, his brain lesion was actually associated with superior performance to normal. However, JR's latencies on both pure pro- and pure anti-saccades tasks were significantly raised and, unlike controls, pro-saccades were not faster than anti-saccades (Fig. 3). So a likely explanation for his reduced error rate on the pure blocks is that it simply reflects a trade-off between speed and accuracy: the slower the response, the greater the accuracy. But the important point here is that JR is capable of making anti-saccades quite accurately. Therefore, these observations suggest that the SEF may not be essential to making ‘internally-guided’ saccadic eye movements *per se* or cancelling reflexive saccades.

The key finding from the first set of experiments was that on *mixed* blocks of trials, where participants had to switch between pro- and anti-saccades, JR was significantly *worse* than controls when required to make pro-saccades. When signalled to make a pro-saccade, he was more likely than healthy controls to make an anti-saccade, despite having significantly longer latencies for pro-saccades than controls, and in the context of no significant difference in latency for pro- and anti-saccades (Fig. 3a). So a speed-accuracy trade-off cannot easily account for this deficit. Significantly more of JR's pro-saccade errors (81%) occurred following an anti-saccade trial (i.e. after a rule switch), and he was four times more likely to make an error when switching from an anti-saccade to a pro-saccade than *vice versa*.

These findings provide further evidence for the potential importance of the SEF in resolving conflict between competing saccadic responses. According to our hypothesis, the SEF plays a crucial role in motor control specifically in situations of oculomotor response conflict (Husain et al., 2003). Thus, the key aspect of SEF function is not the generation of endogenous saccades but rather in controlling response selection when there is competition between internally-generated saccade plans. Importantly, the problems that JR encountered in switching between established (internal) rules for pro- and anti-saccades were asymmetric: he was significantly worse switching from anti- to pro-saccades. One interpretation for this is that the rules for the harder task (executing anti-saccades) were given inappropriate priority in the competition for selection. The conflict on switch trials between the old rule and the new one was not properly resolved, despite longer latencies for both pro- and anti-saccades’ leading JR to make erroneous anti-saccades when he should have switched to making pro-saccades. A similar general problem in selecting between conflicting saccadic responses appears also to explain his difficulty in the arbitrary stimulus–response learning task for saccades.

### 5.2 Arbitrary stimulus–response learning

Previous electrophysiological recording studies by Chen and Wise (1995a, 1995b, 1996, 1997) have implicated the macaque SEF in learning arbitrary stimulus–response associations. These paradigms usually involve the selection of an action amongst competing alternatives and the reinforcement of the stimulus–response linkage via feedback. JR took significantly longer to reach criterion on a task that required learning of arbitrary stimulus–response mappings for saccades, but not for hand movements (Fig. 5). Clearly neither discriminating sensory cues nor understanding the task was a problem because he was able to perform without difficulty in the manual version of the task. Instead the deficit appears to involve the selection of an appropriate motor plan. Again, this does not reflect a fundamental difficulty in executing ‘internally generated’ saccades. JR was eventually able to achieve error-free performance in this associative learning task and, as noted previously, he could perform anti-saccades with great accuracy. Furthermore, in our previous study we reported his response latencies to centrally arrow-cued saccades were indistinguishable from that for controls (Husain et al., 2003).

Patient JR's deficit in the associative learning task appears to reflect problems in a system that controls selection of a new behavioural rule, uses feedback to reinforce a new rule, or a combination of both these factors. The selection deficit difficulty, we propose, arises when JR is required to choose the correct eye movement response from several *conflicting* alternatives. In our previous study we found that JR had extreme difficulty when required to change an ongoing, or partially prepared, oculomotor plan to make a different eye movement response (Husain et al., 2003). We considered such a deficit to reflect a problem in exerting control over saccades in situations of response conflict. In that experiment, the conflict was between two alternative (conflicting) ongoing saccadic responses—the planned eye movement and the new one that he was instructed to change to. Similarly, in the novel associative learning task described in the current paper, JR was required to exert control over saccadic responses to select from four competing possibilities. Interestingly, after the experiment JR reported that he frequently knew which target to look at but seemed unable to prevent himself from making an incorrect eye movement to one of the other locations.

An additional explanation for JR's deficit on the associative learning task is that he experienced difficulties in monitoring the outcome of his responses (Amador et al., 2000; Stuphorn et al., 2000). The output of a system monitoring feedback is potentially crucial in reinforcing the link between a stimulus and the appropriate response (Amador et al., 2000). However, in this particular case, such a view would have to be specific for monitoring the outcome of eye movement responses only, and would not be a general error monitoring system. Moreover, in our previous study, we reported that JR could monitor his errors well on both the change-of-plan and a saccadic rule-reversal task (Husain et al., 2003).

We have also re-analyzed our change-of-plan data from that study, examining the *implicit* effects of error monitoring by comparing saccadic latencies on no-change trials following a change trial versus those that did not follow a change trial. JR's post-change trial response times were slowed (mean difference of 28 ms) to a similar degree as the control participants (mean difference of 32 ms), consistent with previous studies in healthy individuals using the related stop paradigm (Hanes & Carpenter, 1999; Schall, Stuphorn, & Brown, 2002). Additionally, JR was slowed more in trials that followed an unsuccessful attempt to inhibit a planned movement than those in which he succeeded (18 ms versus 36 ms), demonstrating that his performance is affected by the outcome of the previous trial (Hanes & Carpenter, 1999; Schall et al., 2002). Thus, these data show that performance outcome does modulate his subsequent responses and suggest that his oculomotor deficits are unlikely to result from an inability to monitor the outcome of saccadic performance.

### 5.3 The SEF and hypometria in memory-guided saccades

Previous studies have suggested that the SEF – particularly the left SEF in humans – plays a role in the control of memory-guided saccade sequences (Gaymard et al., 1990, 1993; Grosbras et al., 2001; Heide et al., 2001; Lu et al., 2002; Schlag-Rey et al., 1997; Sommer & Tehovnik, 1999). Although JR's saccades were of normal amplitude when the targets remained illuminated he demonstrated a significant hypometria when making an eye movement to a remembered location in the dark (Fig. 7). Importantly, although previous studies in humans have suggested that the SEF is important for correct ordering of saccadic sequences (Gaymard et al., 1990, 1993; Muri, Rivaud, Vermersch, Leger, & Pierrot-Deseilligny, 1995; Tobler & Muri, 2002) we found patient JR had *normal* error rates when required to make a eye movements to a series of remembered locations in the dark.

There are a number of potential methodological differences between our investigation of saccadic sequences and previous studies. First, the emphasis in the current paradigm was on response accuracy, so JR was explicitly instructed to pause at each target location, to match the timing intervals between the previously viewed stimuli. It is possible that a more rapid series of responses might have been associated with sequence errors. Such a manipulation would have reduced the temporal segregation between responses giving less time to prepare each movement and to resolve any conflict between successive responses. (Note that in this task, each of the memorized locations are salient and thus potentially in competition. But, when there are clear pauses between responses, the immediate saccade goal may be the most salient, so much so that the sequential saccade task may not be a good measure of the ability to resolve conflicting saccade commands.) Second, the previous studies were either based on patients with much larger lesions that subsumed other frontal cortical areas (Gaymard et al., 1990, 1993) or supra-threshold TMS which can also effect the haemodynamic response of neurons in adjacent cortical areas (Muri et al., 1995; Tobler & Muri, 2002). Third, Gaymard et al. (1990, 1993) defined incorrect sequences as errors “in chronology, or in number of pauses” which they noted occurred with equal frequency. The later type of error might actually reflect the occurrence of hypometric saccades followed by large corrective movements, rather than a sequence deficit. Finally, the spatial resolution of the tracking equipment used in the current experiment was superior to that in the earlier studies. All of the previous studies contain indications of inaccuracies in saccadic amplitudes which may have been underestimated because of the resolution of the eye tracking system used.

Nevertheless, the hypometria for memory-guided eye movements clearly demonstrates the existence of a deficit in making a planned eye movement to a remembered target location. Similar hypometria has also been documented in humans experiencing threshold levels of TMS to the SEF whilst performing memory-guided saccade sequences (Rosenthal, Offord, Hodgson, & Kennard, 2003). Such a deficit might be explained by a problem in controlling saccades when there is a degree of response uncertainty: in comparison to remembered saccades to illuminated locations, memory-guided saccades in the dark are always associated with a greater degree of uncertainty about target location. A very similar pattern of undershooting in the dark has been observed in patients with Parkinson's disease on exactly the same task (Hodgson et al., 1999). The direct connections of the SEF with the basal ganglia (Parthasarathy, Schall, & Graybiel, 1992), particularly with the caudate nucleus, may be important for the generation of the correct metrics when memory-guided saccadic responses – either sequences or single saccades – have to be produced in the dark.

The hypometria observed for memory-guided saccades was greater for left versus right saccades, but JR's impairments for all other tasks were bilateral, consistent with our previous findings on a change of saccadic plan task and rule-reversal task (Husain et al., 2003). Monkey recording studies have revealed that each SEF encodes the direction of both leftward and rightward saccades, perhaps with some topographical mapping such that contralateral saccades are represented more anteriorly than ipsilateral ones (Tehovnik et al., 2000). Our previous functional imaging localizer study (Husain et al., 2003) had suggested that part of the anterior portion of the SEF might be spared and it is possible this may contribute to some of the asymmetry observed on this paradigm. This deficit for saccades made in the dark to remembered positions may reflect a role for the SEF in oculomotor control in conditions of response uncertainty such as when visual cues are no longer unavailable. However, it is possible that the deficit observed here reflects the loss of an interaction between the SEF and the basal ganglia, since Parkinson's disease patients show a similar deficit, and may not reflect the function of the SEF alone.

The lack of a deficit in executing saccadic sequences in the correct order in JR suggests that the SEF may not be required to resolve potential conflict between eye movement plans when they are made serially with a large temporal segregation. Instead, our findings implicate the SEF as a key structure in implementing control over the oculomotor system in situations of response conflict, when there are several ongoing, competing eye movement plans. This conceptualization of SEF function explains why it appears to play a critical role in oculomotor control when the association between a stimulus and saccadic response has to be changed (as when switching from anti- to pro-saccades); or when the appropriate saccade has to be selected from competing responses with weakly reinforced stimulus–response associations (as in the arbitrary stimulus-learning task); or when an ongoing saccade plan suddenly has to be altered in favour of a new one (as in the change-of-plan data we reported previously).

## Footnotes

1 JR performed a larger number of trials than controls so it is possible that this may have resulted in a greater practice effect on his performance. Analysis of the first and last 25% of trials in each condition revealed that, for most conditions (6/8), there was actually a slight increase in RT between the first and last quartile (i.e., in the opposite direction to predictions based on practice). The differences were small, with the largest difference in the order of ∼0.2S.D.s, and did not approach significance (*p* > 0.1).

2 Nakamura et al. (2005) based their conclusions on the results of two saccadic paradigms. In the first the direction of a saccade (left or right) was signalled by the colour of a cue which appeared abruptly at either the target location (no conflict) or the opposite location (conflict). The second paradigm was a saccadic change-of-plan task similar to that previously reported by Husain et al. (2003). In most trials saccade direction (left or right) was made in accordance with the colour of central cue (no conflict), but on some trials the colour would suddenly change signalling a need to reverse the saccade direction (conflict).
